# Metachronous bilateral ovarian metastasis with spontaneous rupture following curative resection of stage T4a colon cancer: a case report and literature review

**DOI:** 10.3389/fonc.2026.1950347

**Published:** 2026-10-20

**Authors:** Yanwen Zheng, Zequn Zhang

**Affiliations:** Department of General Surgery, Second Xiangya Hospital, Central South University, Changsha, China

**Keywords:** colorectal cancer, metachronous metastasis, ovarian transposition, salpingo-oophorectomy, spontaneous rupture

## Abstract

**Background:**

Colorectal cancer (CRC) metastasizes to the ovary in approximately 0.8–7.4% of cases, typically indicating aggressive biology and poor prognosis. Metachronous bilateral ovarian involvement with spontaneous rupture is exceptionally rare, and the distinction between signet-ring cell (Krukenberg) and non-signet-ring cell metastases carries prognostic significance.

**Case presentation:**

We present a 54-year-old female who underwent laparoscopic right hemicolectomy for pT4aN0M0 ascending colon adenocarcinoma, followed by eight cycles of adjuvant XELOX chemotherapy. Nineteen months later, imaging revealed huge pelvic cystic-solid masses with diffuse peritoneal thickening. Intraoperatively, spontaneous rupture of the mass (~14 cm) with active bleeding and hemorrhagic ascites was found, along with extensive miliary peritoneal seeding. The patient underwent bilateral salpingo-oophorectomy, omentectomy, and HIPEC catheter placement. Pathology confirmed bilateral ovarian metastatic adenocarcinoma of colorectal origin with no signet-ring cells, excluding a true Krukenberg tumor. Genetic testing revealed NRAS p.G12D mutation and high tumor mutational burden (TMB-H). She subsequently received six cycles of XELOX chemotherapy with bevacizumab and serplulimab (anti-PD-1), achieving stable disease.

**Conclusion:**

This case demonstrates that non-signet-ring cell ovarian metastases can be equally aggressive, manifesting as spontaneous rupture after standardized therapy. For T4a CRC patients with serosal invasion, vigilant surveillance for ovarian and peritoneal metastasis is warranted. The multimodal strategy of cytoreductive surgery, HIPEC, and genotype-directed immunotherapy may offer a promising salvage approach in this challenging setting.

## 1 Introduction

Colorectal cancer (CRC) is the third most common malignancy worldwide, with the liver and peritoneum being the predominant sites of metastasis. Ovarian involvement, though less frequent (occurring in 0.8%–7.4% of female CRC patients), represents a particularly unfavorable prognostic event, with a median survival often less than 20 months (1, 2). The route of spread is believed to be primarily transcoelomic (peritoneal seeding), hematogenous, or lymphatic, with serosal invasion (pT4) being a critical risk factor (3).

A significant pathological distinction exists within ovarian metastases: the Krukenberg tumor, defined strictly by the presence of >10% signet-ring cells within a cellular ovarian stroma, versus non-signet-ring cell metastases, which retain the glandular architecture of the primary adenocarcinoma (4, 5). While Krukenberg tumors are notoriously chemoresistant and aggressive, the biological behavior of non-signet-ring cell metastases is less clearly defined, with some cases exhibiting equally rapid progression. Spontaneous rupture of ovarian metastases leading to acute hemoperitoneum is exceedingly rare, with only sporadic case reports available (6–9). Furthermore, the optimal systemic therapy after failure of standard platinum/oxaliplatin-based adjuvant regimens—especially in the context of actionable mutations like NRAS and high tumor mutational burden (TMB-H)—remains an area of active exploration.

We report a unique case of metachronous bilateral non-signet-ring cell ovarian metastasis with spontaneous rupture occurring 19 months after curative resection and adjuvant XELOX for pT4a colon cancer, successfully managed with cytoreductive surgery, HIPEC, and subsequent triplet therapy comprising chemotherapy, bevacizumab, and an immune checkpoint inhibitor. This case underscores the aggressive potential of non-Krukenberg ovarian metastases and highlights a genotype-driven multidisciplinary management strategy.

## 2 Case presentation

A 54-year-old female, with a past surgical history of total hysterectomy for uterine fibroids in 2016, underwent laparoscopic right hemicolectomy for ascending colon cancer in July 2024 at an outside tertiary hospital. Postoperative pathology (pT4aN0M0, Stage IIB) revealed the tumor invaded through the visceral peritoneum (pT4a), with perineural invasion identified; all 23 harvested lymph nodes were negative (pN0) (**Table 1**). The patient completed eight cycles of adjuvant XELOX chemotherapy (oxaliplatin 130 mg/m^2^ day 1, capecitabine 1000 mg/m^2^ BID days 1–14, q3w) between August 2024 and March 2025. After completing adjuvant chemotherapy in March 2025, the patient was advised to undergo surveillance every 3 months with clinical examination, serum tumor markers (CEA, CA19-9, CA125), and abdominopelvic ultrasound, as well as contrast-enhanced CT of the chest, abdomen, and pelvis every 6 months. She attended follow-up visits in June 2025 and September 2025, at which she was asymptomatic, with normal serum CEA and CA19–9 levels and no pelvic abnormalities on ultrasound. However, she did not return for subsequent scheduled visits due to personal reasons. She remained asymptomatic until February 2026, when she presented with progressive lower abdominal distension.

**Table 1:** Pathological, immunohistochemical, genetic characteristics of the primary tumor and metachronous ovarian metastases.

Specimen	Pathology	Immunohistochemistry (IHC)	Genetic testing
First surgery (primary tumor, Aug 2024)	TNM stage: pT4aN0M0 Histology: Moderately to well-differentiated adenocarcinoma (ulcerative type), with intermediate tumor budding, measuring 2.5 × 2.5 × 0.7 cm. Serosal layer was invaded. Lymph nodes: 0/23. Omentum: No metastasis.	BRAF V600E (-) PD-L1 (SP263, Ventana): CPS < 1 HER2 (C-erbB-2): 0 MMR proteins: MSH2 (+), MSH6 (+), MLH1 (+), PMS2 (+) → pMMR CK (+), CDX2 (+), S100 (perineural invasion confirmed) TROP2 (focal +), Ki-67: 50%	Not performed
Second surgery (ovarian metastases, Mar 2026)	Specimens: Two cystic-solid masses from bilateral adnexa, measuring 8×7×5 cm and 14×9×7 cm, with thin external capsules and containing yellow fluid. No normal ovarian tissue was identified. Diagnosis: Bilateral ovarian metastatic adenocarcinoma, consistent with colorectal origin.	Ki-67: 70% p53: mutant-type expression SATB2: (partial +),PAX-8(-) HER2 (C-erbB-2): 1+ CA125 (-), WT-1 (-), p16: (patchy +), ER: 0, PR: 0, CDX2 (+) MMR proteins: MLH1 (+), MSH2 (+), MSH6 (+), PMS2 (+) → pMMR	Somatic variants: 8 variants detected, including two clinically actionable: *NRAS* p.G12D and *TP53* p.C176R. Germline variants: None detected. MSI: Microsatellite stable (MSS). TMB:8.33muts/Mb→TMB-H (high). ICI-related gene: *TP53* p.C176R (positively correlated with response).

CPS, combined positive score; pMMR, proficient mismatch repair; MSS, microsatellite stable; TMB-H, high tumor mutational burden; ICI, Immune checkpoint inhibitor.

In February 2026—approximately 19 months after her initial surgery—the patient presented with progressive lower abdominal distension and mild discomfort. Local hospital ultrasonography revealed bilateral adnexal masses. Serum tumor markers were markedly elevated: CEA 188.25 ng/mL (ref: <5.0), CA19-9 462.77 U/mL (ref: <35.0), CA125 303.91 U/mL (ref: <35.0), and CA72-4 21.95 U/mL (ref: <6.9).

She was referred to our institution in March 2026. Contrast-enhanced abdominopelvic CT revealed peritoneal thickening and multiple soft-tissue pelvic masses, the largest measuring 94 × 73 mm, with involvement of the bladder and rectal walls (**Figure 1**). Colonoscopy showed no anastomotic recurrence. Whole-body ^18^F-FDG PET-CT demonstrated several cystic-solid left pelvic masses with heterogeneous hypermetabolism, indistinct from both adnexa (suspicious for malignancy; Krukenberg tumor was radiologically suspected, but primary ovarian malignancy could not be excluded) (**Figure 2**). Diffuse peritoneal thickening with increased FDG uptake and perihepatic/perisplenic ascites were consistent with peritoneal carcinomatosis.

**Figure 1:**
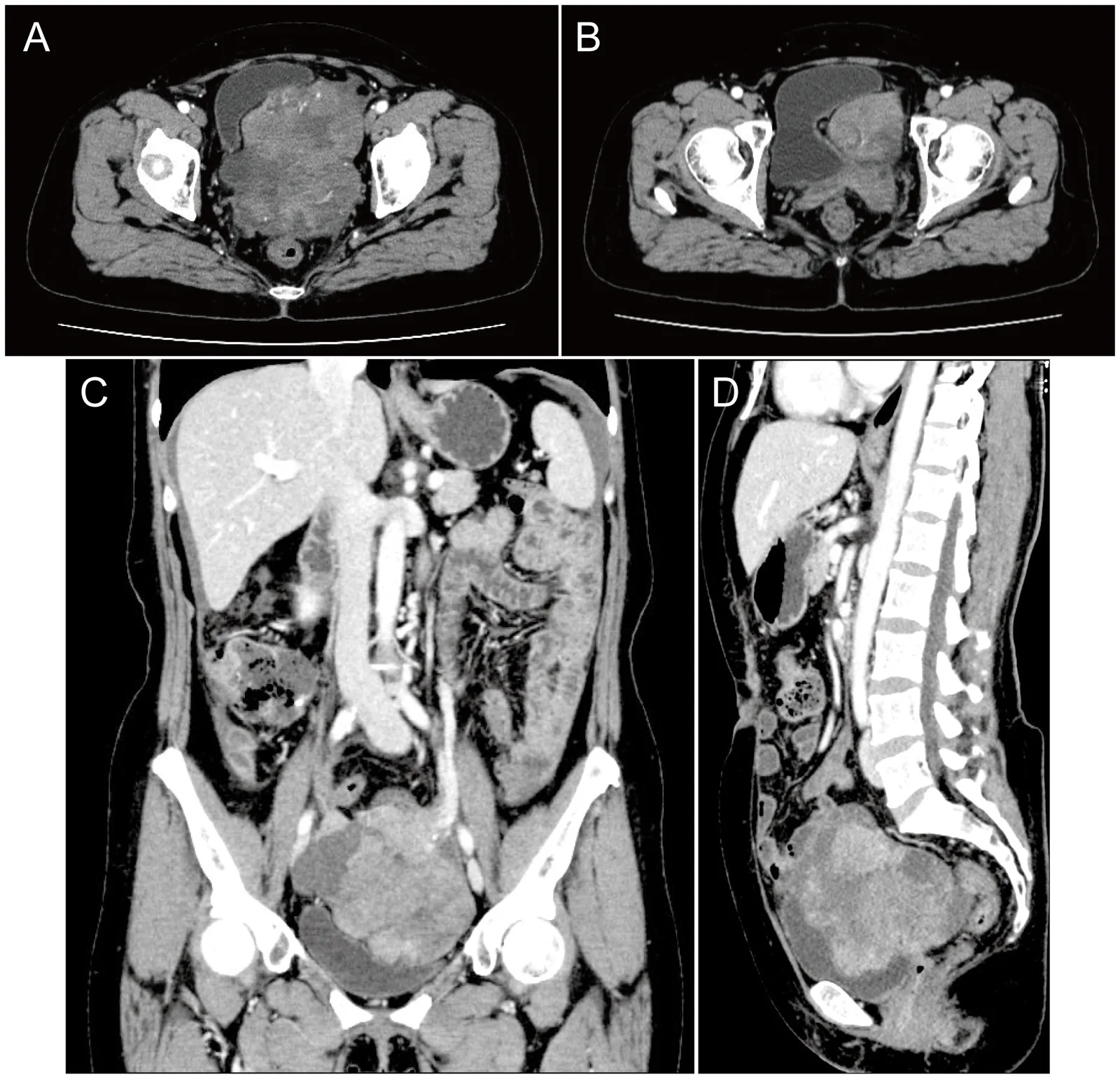
Preoperative contrast-enhanced computed tomography (CT) images of the pelvic masses. **(A, B)** Axial CT images demonstrating large pelvic masses with heterogeneous enhancement. **(C, D)** Coronal and sagittal reformatted images, respectively, showing that the tumors almost completely occupied the pelvic cavity.

**Figure 2:**
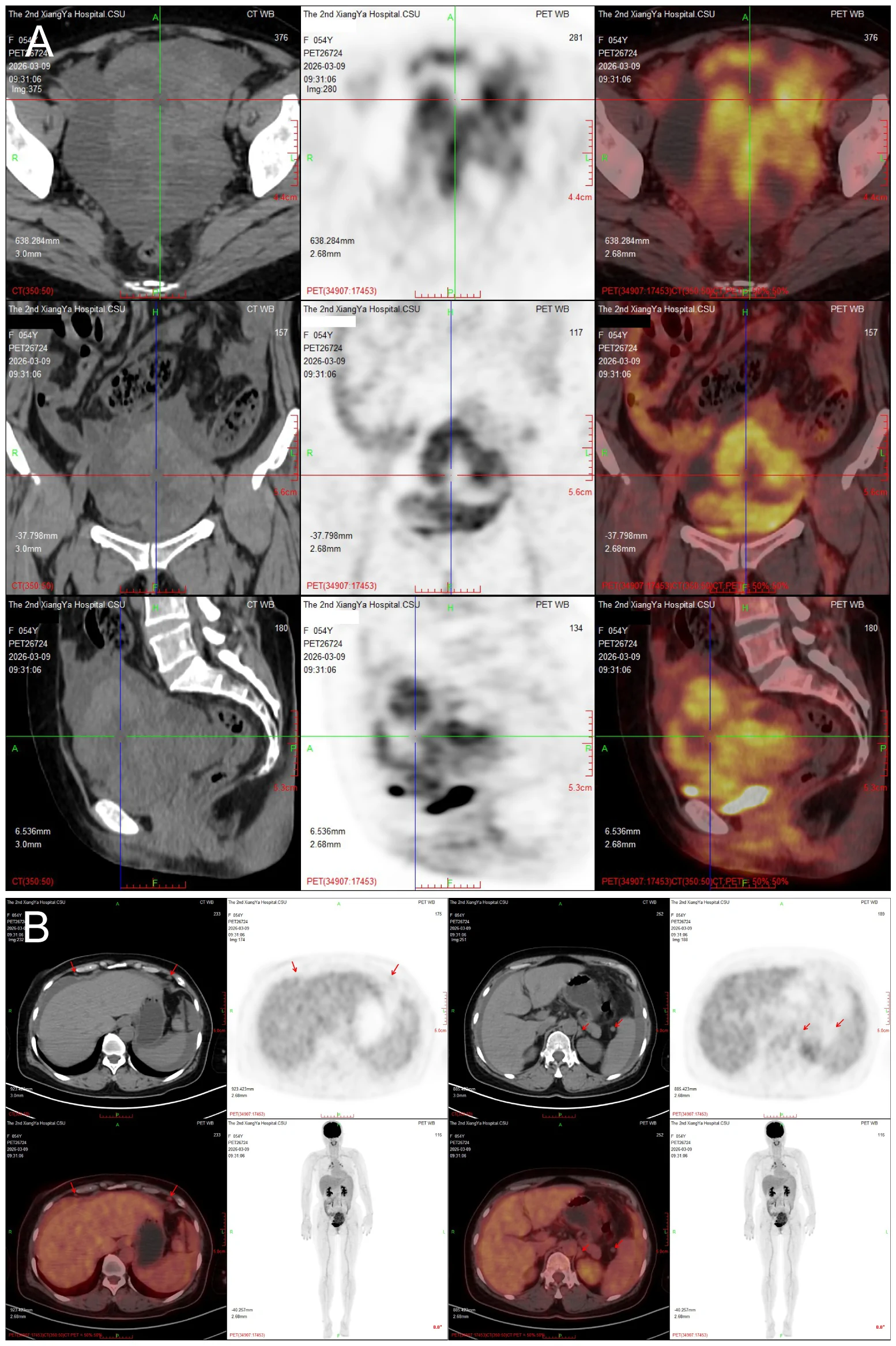
Preoperative ^18^F-fluorodeoxyglucose (FDG) positron emission tomography-computed tomography (PET-CT) images. **(A)** A huge cystic-solid mass with heterogeneous increased glucose metabolism in the left pelvis. **(B)** Diffuse peritoneal thickening with increased FDG uptake and perihepatic/perisplenic fluid collections.

A multidisciplinary team (MDT) consultation, comprising radiologists, gastrointestinal surgeons, gynecologic oncologists, anesthesiologists, and medical oncologists, concluded that the pelvic masses most likely represented ovarian metastases from colorectal cancer. The diagnosis was considered recurrent colon cancer with ovarian metastasis, with a pelvic ovarian metastatic mass exceeding 14 cm. The tumor burden was large and posed several potential risks, including spontaneous rupture and hemorrhage, bowel obstruction due to compression, and, if surgery were delayed, the potential loss of the optimal window for elective surgery. Although PET-CT showed diffuse peritoneal thickening with increased FDG uptake suggestive of peritoneal carcinomatosis, the dominant lesions were confined to the pelvis and were considered potentially resectable. Therefore, the MDT recommended cytoreductive surgery with possible bowel resection, followed by systemic therapy, rather than a purely palliative procedure. The patient was considered a candidate for maximal cytoreduction, although complete cytoreduction was not anticipated preoperatively due to the known peritoneal involvement. Notably, although tumor rupture was not detected preoperatively, it was found immediately upon abdominal entry in the late surgery; we therefore postulate that the tumor may have ruptured suddenly at some point preoperatively. This further supported the decision not to delay surgery.

The patient underwent surgery in March 2026. Upon entering the abdominal cavity, a life-threatening finding was encountered: massive hemorrhagic ascites gushed out, and the region of ovaries was replaced by a huge, tense, cystic-solid mass, approximately 14 cm in diameter, with active bleeding from surface rupture sites (**Figure 3**). The fallopian tubes were indistinct, and the uterus was absent. After urgent hemostasis and aspiration of all intraperitoneal fluid, bilateral salpingo-oophorectomy, omentectomy, and HIPEC catheter placement were performed. Exploration also revealed extensive miliary peritoneal seeding nodules throughout the abdomen. Complete cytoreduction was not achieved due to the extensive peritoneal seeding; residual disease remained (CC-2/3). The prior right colon anastomosis was intact without obstruction or local recurrence. Postoperative pathology confirmed bilateral ovarian metastatic adenocarcinoma consistent with colorectal origin, supported by immunohistochemistry (CK7-/CK20+/CDX2+) (**Figure 3**). Notably, no signet-ring cells were identified, thus excluding the diagnosis of a true Krukenberg tumor. Extensive peritoneal implantation was confirmed.

**Figure 3:**
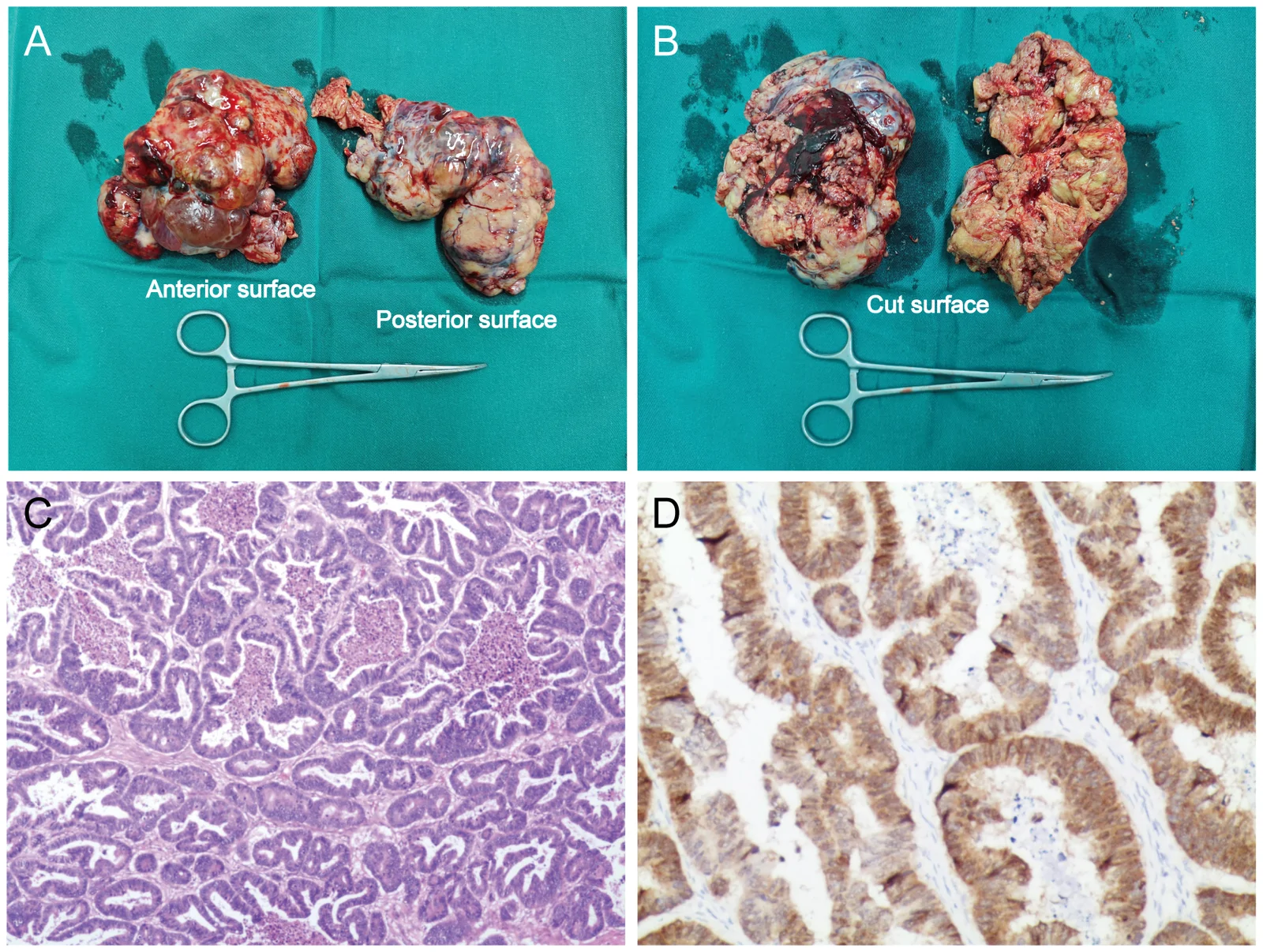
Macroscopic and histopathological features of the resected tumor. **(A)** Gross appearance of the bisected tumor specimen showing the overall morphology. **(B)** Cut surface of the tumor revealing a cystic-solid architecture. **(C)** Hematoxylin and eosin (H&E) staining of the tumor tissue (original magnification, ×40). **(D)** Immunohistochemical staining for CDX2 showing nuclear positivity, supporting colorectal origin (original magnification, ×100).

The patient received three sessions of HIPEC on postoperative days 1, 3, and 5 (sterile water, raltitrexed 4 mg, and oxaliplatin 200 mg, respectively, the total volume of perfusate for each HIPEC session was 4000 mL). This sequential, multi-agent regimen was selected based on our center’s experience with HIPEC for peritoneal carcinomatosis of colorectal origin and on the patient’s tolerance, representing an individualized treatment approach. Each perfusion was performed at a target intraperitoneal temperature of 42–43 °C for 60 minutes. Raltitrexed was selected based on preclinical evidence of hyperthermia synergism in colorectal cancer organoids, and oxaliplatin was chosen for its established activity in colorectal peritoneal carcinomatosis. The intraperitoneal catheters were removed on postoperative day 7 after completion of the final HIPEC session, once the drainage fluid was clear and the daily drainage volume was less than 100 mL. The patient was surveilled for postoperative complications for 90 days after cytoreductive surgery. During this period, no major postoperative complications occurred. According to the Clavien–Dindo classification, the postoperative course was graded as 0–I. There were no events of anastomotic leakage, intra-abdominal infection, postoperative hemorrhage, bowel obstruction, or catheter-related complications requiring reintervention. The patient recovered smoothly and remained clinically stable throughout the surveillance period. Postoperative genetic profiling of the ovarian metastasis revealed an NRAS p.G12D mutation and high tumor mutational burden (TMB-H). After MDT consultation, systemic triplet therapy was initiated: XELOX chemotherapy, plus bevacizumab and serplulimab (an anti-PD-1), repeated every 3 weeks. At the time of this report, the patient has completed six cycles of this combined regimen. Imaging and tumor marker assessments demonstrate stable disease, and she continues regular follow-up.

## 3 Discussion

We report a case of metachronous bilateral ovarian metastasis with spontaneous rupture following apparently curative resection of a T4a colon cancer. Our case carries several pivotal teaching points.

First, it highlights the aggressive biology of non-signet-ring cell ovarian metastases. Pathologically, a true Krukenberg tumor is defined by the presence of signet-ring cells occupying >10% of the tumor. The absence of these cells in our patient means this is a non-signet-ring cell ovarian metastasis. Literature suggests that while Krukenberg tumors are associated with exceptionally poor outcomes, non-signet-ring cell metastases are often considered slightly less aggressive (5, 10); however, our case challenges this assumption. The tumor displayed rapid growth (reaching 14 cm), bilateral involvement, and spontaneous rupture—behaviors typically ascribed to the most virulent neoplasms. This underscores that any colorectal ovarian metastasis, regardless of histologic subtype, can exhibit profound chemoresistance and lethality (2).

Second, the spontaneous rupture of ovarian metastases is an exceedingly rare but life-threatening event. While uterine or vaginal bleeding may occasionally occur in patients with ovarian metastasis through hormonal or mechanical mechanisms, it is neither a specific nor a consistent early warning sign. In our patient, who had previously undergone total hysterectomy, the uterus and its potential bleeding-related symptoms were absent. This likely allowed the tumor to grow silently to a massive size without early clinical detection, eventually exceeding capsular tension limits and presenting acutely with rupture and hemoperitoneum. Only a handful of cases have reported ovarian metastasis rupture (6–9). This emphasizes that in patients with colorectal cancer—particularly those with a history of hysterectomy—the possibility of ruptured ovarian metastasis should be considered when acute abdomen with sudden-onset shock occurs, and urgent surgical intervention is warranted. To contextualize our case within the existing literature and to highlight the distinctive features of our patient, we summarized reported cases of metachronous ovarian metastasis from colorectal cancer following curative primary resection without initial distant metastasis (**Table 2**). A chronological review of these cases reveals several important patterns. On one hand, the interval from primary surgery to ovarian metastasis ranged widely, from 12 months to 3 years, with the majority occurring within 2 years, consistent with prior reports. On the other hand, while some patients presented with solitary unilateral ovarian involvement without peritoneal disease, our case demonstrated bilateral ovarian masses with extensive peritoneal seeding, which is more commonly associated with aggressive tumor biology. Moreover, the spontaneous rupture of ovarian metastasis observed in our case is an exceedingly rare event, with no similar presentation identified in the summarized cases.

**Table 2:** Summary of reported cases of metachronous ovarian metastasis from colorectal cancer following curative primary resection.

Author, year	Age	Primary tumor (site, stage)	Primary therapy (surgery + adjuvant)	Inter-val	Metastasis details(size, laterality, rupture, peritoneal)	Pathological type (SRC/non SRC)	Treatment for metastasis	Outcome
Sahai et al., 2014 (11)	17	Transverse colon, pT3N1M0	Right hemicolectomy + FOLFOX ×11	23m	ilateral ovarian masses (~9 cm), pelvic/uterine/rectal infiltration, peritoneal deposits	Mucinous adenocarcinoma (CK20+/CK7−)	Chemotherapy (irinotecan + capecitabine) → TAH + BSO → pelvic radiotherapy	Progressive disease (retroperitoneal LNs); symptom-free at 26 months post-KT diagnosis
Paramythiotis et al., 2019 (12)	66	Sigmoid colon, pT3N0M0	Left hemicolectomy, no adjuvant therapy	3yr	right ovary (8.6 × 5.6 cm), no peritoneal involvement	Adenocarcinoma (CK20+/CDX2+/CK7−)	TAH + BSO	Referred for adjuvant treatment
Bodofsky et al., 2021 (13)	29	Sigmoid colon, pT4bN1b	Low anterior resection + left SO + FOLFOX ×10 + pelvic chemoradiation	~12m	right transposed ovary (8.0 × 6.5 × 6.3 cm), no peritoneal involvement	Adenocarcinoma (CK20+/CDX2+/CK7−/PAX8−)	Right oophorectomy	Not specified
Almas et al., 2020 (14)	19	Appendix/colon	Appendectomy → right hemicolectomy + chemotherapy ×6	~2yr	left adnexal mass (26.5 × 14.7 × 34 cm), widespread dissemination at recurrence	Metastatic colon adenocarcinoma	Left radical SO + omentectomy → chemotherapy	Widespread dissemination at 6 months; patient deceased
Tang et al., 2024 (15)	48	Rectal/colonic origin	CRC surgery + FOLFOX ×3 (patient refused further follow-up)	14m	right ovary (13 × 12 × 12 cm), solitary, no peritoneal involvement	Metastatic adenocarcinoma (CK20+/CDX2+/CK7−)	TAH + BSO	Disease-free at 18 months post-metastasectomy
Our case, 2026	54	Ascending colon, pT4aN0M0	Laparoscopic right hemicolectomy + XELOX ×8	19m	Bilateral (~12 cm on one side), spontaneous rupture, extensive peritoneal seeding	Non-signet-ring cell (CK20+/CDX2+/SATB2+)	CRS (BSO + omentectomy) + HIPEC + XELOX/Bev/Serplulimab	Stable disease (6 cycles)

Bev, bevacizumab; BSO, bilateral salpingo-oophorectomy; CRC, colorectal cancer; CRS, cytoreductive surgery; FOLFOX, 5-FU, Leucovorin, and Oxaliplatin; HIPEC, hyperthermic intraperitoneal chemotherapy; KT, Krukenberg tumor; LN, lymph node; SO, salpingo-oophorectomy; SRC, signet-ring cell; TAH, total abdominal hysterectomy; XELOX, capecitabine plus oxaliplatin.

Third, our case underscores the clinical significance of the pT4a stage. Despite negative lymph nodes (pN0), serosal invasion allows tumor cells to shed directly into the peritoneal cavity (transcoelomic spread). This pathway provides a direct route to the ovaries and peritoneal surfaces, circumventing the lymphatic system (3). The 19-month interval to recurrence is consistent with reported data indicating that metachronous ovarian metastases usually occur within 2 years of primary resection (1). Our case emphasizes the need for heightened vigilance for ovarian and peritoneal metastasis in patients with T4a colorectal cancer, irrespective of nodal status or histological subtype.

Fourth, the presence of an NRAS p.G12D mutation and TMB-H guided our salvage therapy. RAS mutations are known to confer resistance to anti-EGFR therapies but may still respond to anti-angiogenic agents like bevacizumab (16). TMB-H is a well-established biomarker for responsiveness to PD-1/PD-L1 inhibitors (17). The combination of chemotherapy, bevacizumab, and serplulimab (anti-PD-1) represents a rational, synergistic strategy. This approach is supported by the phase 2 part of the randomized ASTRUM-015 trial, in which first-line serplulimab plus a bevacizumab biosimilar and XELOX significantly prolonged median PFS compared with placebo plus bevacizumab and XELOX (17.2 vs. 10.7 months; HR 0.60), with consistent benefit in the MSS subgroup (17.2 vs. 10.1 months; HR 0.58) and the KRAS-mutant subgroup (17.2 vs. 10.1 months; HR 0.40) (18). A recent real-world case report also described a patient with MSS/KRAS p.G12D-mutant metastatic colon cancer treated with the same triplet who achieved a clinical complete response and remained progression-free beyond 12 months (19). The combination of chemotherapy, bevacizumab, and serplulimab (anti-PD-1) represents a rational, synergistic strategy. Chemotherapy and anti-angiogenesis can modulate the tumor immune microenvironment, potentially enhancing the efficacy of immune checkpoint blockade (20). Our patient’s achievement of stable disease after six cycles suggests this regimen may be a viable option for this molecularly defined subgroup, though longer follow-up is required. We acknowledge that this triplet regimen was not yet incorporated into standard guidelines, that the NRAS-mutant subgroup in ASTRUM-015 was very small, and that the PD-1 inhibitor was subsequently held in our patient due to an immune-related adverse event; the decision was therefore individualized and made through multidisciplinary discussion.

Finally, the multidisciplinary approach—from accurate radiological diagnosis to surgical cytoreduction, HIPEC, and genotype-matched systemic therapy—is the cornerstone of managing such complex cases (21). Our experience suggests that even in the setting of widespread peritoneal disease and acute rupture, aggressive local control (CRS+HIPEC) combined with modern systemic therapy can achieve disease stabilization.

Several limitations of this report should be acknowledged. First, the findings are derived from a single case, which limits their generalizability. The clinical course and treatment response observed in this patient cannot be extrapolated to all patients with colorectal ovarian metastasis, and the favorable outcome may not be reproducible in other clinical settings. Second, the role of HIPEC in this specific context remains uncertain. Although HIPEC is used in selected patients with peritoneal carcinomatosis from colorectal cancer, the phase 3 PRODIGE 7 trial did not demonstrate a significant overall survival benefit when HIPEC was added to complete cytoreductive surgery. Therefore, the contribution of HIPEC to the favorable outcome in our patient cannot be determined, and its use should be regarded as individualized and investigational. Third, the follow-up duration is relatively short, and long-term survival outcomes remain unknown. Finally, the triplet regimen including serplulimab was based on emerging evidence and was subsequently modified because of an immune-related adverse event; the optimal duration and sequencing of immunotherapy in this molecular subgroup require further prospective study. These limitations should be considered when interpreting the findings of this report.

In conclusion, we present a rare case of metachronous bilateral non-signet-ring cell ovarian metastasis with spontaneous rupture after standardized adjuvant therapy for pT4a colon cancer. This case demonstrates that absence of signet-ring cells does not guarantee indolent behavior; these metastases can be equally aggressive and prone to catastrophic complications. Clinicians should maintain a high index of suspicion for ovarian involvement in female CRC patients with serosal invasion, regardless of nodal status. Furthermore, the combination of cytoreductive surgery, HIPEC, and subsequent triplet therapy (chemotherapy + bevacizumab + PD-1 inhibitor) guided by NRAS and TMB status appears promising and warrants further investigation in larger studies. The patient continues to respond well to this regimen, and ongoing surveillance is crucial to assess long-term survival outcomes.

## Data Availability

The original contributions presented in the study are included in the article/supplementary material. Further inquiries can be directed to the corresponding author.
